# Sex Differences Are Here to Stay: Relevance to Prenatal Care

**DOI:** 10.3390/jcm10133000

**Published:** 2021-07-05

**Authors:** Amy M. Inkster, Icíar Fernández-Boyano, Wendy P. Robinson

**Affiliations:** 1 BC Children’s Hospital Research Institute, Vancouver, BC V5Z 4H4, Canada; ainkster@bcchr.ca (A.M.I.); iciar.fernandez@bcchr.ca (I.F.-B.); 2 Department of Medical Genetics, University of British Columbia, Vancouver, BC V6H 3N1, Canada

**Keywords:** sex as a biological variable, sex differences, pregnancy complications, placenta, prenatal diagnosis, preeclampsia, preterm birth, fetal growth restriction, miscarriage

## Abstract

Sex differences exist in the incidence and presentation of many pregnancy complications, including but not limited to pregnancy loss, spontaneous preterm birth, and fetal growth restriction. Sex differences arise very early in development due to differential gene expression from the X and Y chromosomes, and later may also be influenced by the action of gonadal steroid hormones. Though offspring sex is not considered in most prenatal diagnostic or therapeutic strategies currently in use, it may be beneficial to consider sex differences and the associated mechanisms underlying pregnancy complications. This review will cover (i) the prevalence and presentation of sex differences that occur in perinatal complications, particularly with a focus on the placenta; (ii) possible mechanisms underlying the development of sex differences in placental function and pregnancy phenotypes; and (iii) knowledge gaps that should be addressed in the development of diagnostic or risk prediction tools for such complications, with an emphasis on those for which it would be important to consider sex.

## 1. Introduction

Sex differences exist throughout the life course, with the earliest differences evident well before birth and spanning gestation. Pregnancies carrying male and female fetuses may differ in their risks of early pregnancy loss, preterm birth, and placental insufficiency associated with preeclampsia and/or fetal growth restriction. However, establishing the influence of sex on these outcomes is complicated by the different diagnostic criteria and genetic and environmental risk factors in the populations studied. As the placenta mediates fetal growth and underlies many pregnancy complications, sex differences arising in gestation are likely due to effects of sex on placental development and function. Compared to females (XX), male (XY) fetuses are larger by the second trimester of pregnancy (based on ultrasound data) [1,2], show a more pro-inflammatory immune response across gestation, and are at a higher risk of infection leading to preterm birth and other pregnancy complications [3,4,5]. This in turn may contribute to sex differences in early susceptibility to childhood conditions including neurodevelopmental disorders [6,7,8]. Throughout life, females remain at lower risk of infection, but are more likely than males to develop adult-onset autoimmune diseases [9]. Sex differences extend well beyond steroid hormones, reproductive organs, and body size; sex differences also affect factors such as disease incidence, and are of value to consider with respect to diagnostic criteria and therapeutic efficacy [10]. 

**Figure 1:**
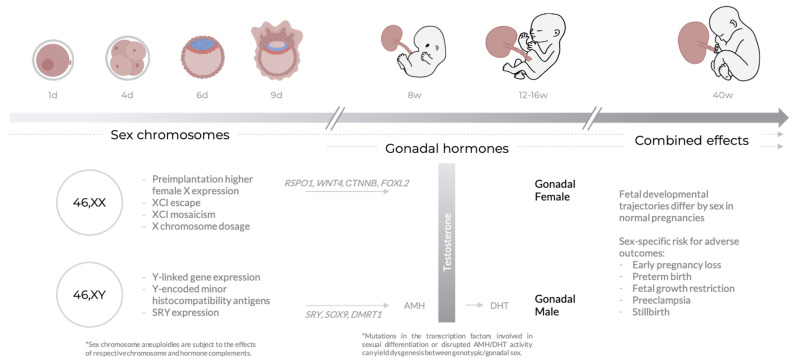
Typical sex differences across gestational age. Throughout pregnancy, sex differences may arise as a consequence of both sex chromosome and sex hormone (testosterone) biology. The combined effects of sex chromosomes and hormones on placental function may contribute to sex differences in healthy development and risk for adverse pregnancy outcomes. Section 3 contains more detailed descriptions of the processes mentioned in this figure, in particular those relating to the X chromosome.

Characterizing the mechanisms that underlie sex differences observed in pre- and perinatal complications may contribute to our understanding of why these sex differences are observed, including the key pathways involved, and has the potential to lead to more effective sex-informed diagnostic and therapeutic practices. Fetal sex steroid hormone production begins partway through the first trimester [11], and therefore sex differences arising earlier in gestation are likely to be due to differential expression of genes on the sex chromosomes, or other sex chromosome effects. Later in development, sex differences may be influenced by transient higher testosterone levels produced by the male fetal testes between 12 and 16 weeks of gestation [12,13,14]. Importantly, sex differences are generally not discrete: for example, testosterone levels and fetal size measurements show considerable variation within each sex, and measurements can overlap between the sexes. In this review, we discuss the sex differences observed in common pregnancy complications, discuss the underlying mechanisms that may be involved, and emphasize the need for collection of fetal sex-specific data when assessing diagnostic and screening tools aimed at promoting healthy birth outcomes (Figure 1).

Sex differences arise as consequences of the processes of sex determination and differentiation; for more information on these processes, see [15,16]. In XY embryos, gonadal upregulation of sex-determining *SRY* initiates a gene expression cascade, leading to sexual differentiation. *SRY* activates *SOX9*, which triggers testis differentiation pathways including the upregulation of *AMH* (anti-Mullerian hormone), leading to regression of the Mullerian ducts. In males, expression of *DMRT1* is also required to antagonize female differentiation pathways. Testosterone, produced by the Leydig cells of the male testes after internal differentiation, is oxidized to the more potent dihydroxytestosterone (DHT), which induces differentiation of the male external genitalia. In the female, lack of *SRY* expression enables upregulation of *RSPO1* and *WNT4*, which cooperatively upregulate *CTNNB* (coding for β-catenin) and accordingly inhibit *SOX9* expression; this allows for the differentiation of the Mullerian ducts into the female reproductive tract. β-catenin also activates *FOXL2* to further repress male differentiation factors including *SOX9*. Mutations in these important transcription factors, or in several other transcription factors involved in downstream gene regulatory networks leading to sexual differentiation, have the potential to lead to gonadal dysgenesis, a spectrum of conditions in which the gonads develop out of accord with genetic sex.

## 2. Sex Differences in Prenatal and Perinatal Complications 

Although an increased male vulnerability to several adverse pregnancy outcomes and complications is well recognized [17], the so-called “male disadvantage” is not consistent across pregnancy complications or throughout gestation. Recent work suggests that although male mortality is elevated in later pregnancy, the opposite is true in early gestation [18]. The notion of a “fragile sex”, whether male or female, is likely an oversimplification, as pregnancy complications differ in their multifactorial etiologies and underlying mechanisms. Furthermore, variation exists in diagnostic criteria for pregnancy complication across institutions. As many adverse pregnancy outcomes have been associated with abnormal placentation, this review will focus on sex differences in perinatal complications associated with placental insufficiency.

### 2.1. Early Pregnancy Loss 

Worldwide, sex ratio at birth is consistently biased toward males [19]. The sex ratio at conception appears to be balanced [18], which suggests preferential loss of female conceptuses during implantation or early development. Approximately 10% of clinically recognized pregnancies [20,21,22,23] and ~30% of all pregnancies [21,22,23] are spontaneously lost in the first trimester, referred to as early pregnancy loss (EPL), thought to arise from placentation failure secondary to other factors. Studies on sex biases in pregnancy loss have been conflicting. An excess of females has been observed among karyotypically normal spontaneous losses during the first two trimesters [18,24,25,26]; however, such bias can also result from maternal contamination confounding cytogenetic analysis of products of conception [27]. Indeed, other studies have reported that male conceptuses are more susceptible to both early and late pregnancy loss [28,29,30,31], suggesting that female embryos may be preferentially lost during implantation, prior to the detection of pregnancy.

Most sporadic pregnancy losses occur prior to the identification of fetal sex during the routine second trimester anatomy ultrasound [32,33], so early fetoplacental sex data are largely limited to referral for prenatal genetic testing [34,35] or karyotyping of products of conception after miscarriage. Karyotyping after miscarriage is not routine practice, and as such is often limited to cases of recurrent miscarriage (RM), defined as the loss of three of more consecutive pregnancies [36]. Chromosomal abnormalities are associated with approximately 50% of all pregnancy losses [37,38], and with most cases of EPL [39]. In some cases, the fetus carries normal diploid cells while the chromosomal abnormality is confined to the placenta (confined placental mosaicism, CPM), which may allow progression of a pregnancy to term that might otherwise result in early loss [40]. 

Intriguingly, it appears that mosaic trisomy may be more likely to persist to term in females. CPM for trisomy 16, typically originating from trisomy rescue and diagnosed at 10–12 weeks gestational age by chorionic villus sampling (CVS), shows a strong female bias [41]. This could indicate that CPM16 female pregnancies are more resistant to EPL or that mosaicism arises more often in female embryos, though the underlying mechanism is unknown. Similarly, a female preponderance is observed in trisomy 18 cases surviving to term [42,43], which is also associated with placental mosaicism [44], as well as in mosaic trisomy 21 [45,46]. Thus, the susceptibility of either sex to pregnancy loss may be dependent on both the underlying cause and gestational age. Given the importance of chromosomal abnormalities in pregnancy loss, the apparent female bias for prolonged survival of mosaic trisomy pregnancies deserves further study.

### 2.2. Preterm Birth 

Preterm birth (PTB), defined as a live birth prior to 37 weeks of gestation, is a major cause of neonatal morbidity and mortality, and of life-long health complications [47,48]. As with many other pregnancy complications, PTB disproportionately affects individuals of lower socioeconomic status and/or living in lower-average-income countries. Spontaneous PTB is the result of preterm labor with either intact membranes or following preterm premature rupture of membranes (PPROM) [49]. This can arise from a myriad of pathologic processes including infection and decidual senescence. In contrast, iatrogenic PTB is usually indicated by maternal and fetal complications such as preeclampsia and/or fetal growth restriction [49].

Pregnancies carrying a male fetus have a higher incidence of spontaneous PTB independent of other risk factors [5,50,51,52,53,54,55,56,57] (Table 1). Stratification of analyses by gestational age has revealed that male prevalence in spontaneous PTB is greater at earlier gestational ages [5,54,56,58,59]. The trophoblast in male-bearing pregnancies shows a greater pro-inflammatory response to infection, which may contribute to an increase in early spontaneous PTB. Higher rates of spontaneous PTB in male-bearing pregnancies may also indicate a mechanistic link between fetal sex and labor-inducing processes [60]. As opposed to spontaneous PTB, a male excess is not observed for iatrogenic PTB [5,54,56]. This may be explained by the lack of male excess in pregnancy complications that commonly lead to iatrogenic PTB. Either no sex bias or an underrepresentation of males is observed in early iatrogenic PTB (<28 weeks) [59,61,62], most often indicated for preterm preeclampsia [58,59], although this effect may depend on statistical methods used [62]. Therefore, sex differences observed in PTB may differ according to the clinical etiology, and sex differences observed in iatrogenic PTB may further depend on the underlying cause. In addition, preterm males and females also differ in their postnatal clinical course. Morbidities associated with PTB such as bronchopulmonary dysplasia, intraventricular haemorrhage, and infection consistently occur at higher rates in PTB males compared to their female counterparts in various populations [63,64,65]. Moreover, even significant improvements in neonatal care have not narrowed the gap between males and females for neonatal morbidity [66].

It is important to note that many studies of sex biases in PTB have been limited to predominantly white populations, and both genetic risk variants and predisposing environmental risk factors may vary in other populations. As such, these findings may not generalize to all pregnancies; for instance, male excess in spontaneous PTB is insignificant in high-risk pregnancies, where competing risk factors of larger effect may mask the predisposing risk of carrying a male [67]. In addition, while several studies have reported the absence of a male excess among spontaneous PTB in Black and Australian Indigenous populations [51,54,68], other studies disagree [69,70]. It is vital to consider ancestry, ethnicity, and socioeconomic factors when studying the impact of fetoplacental sex on pregnancy complications. 

### 2.3. Fetal Growth Restriction

Fetal growth restriction (FGR) is the condition in which a fetus does not reach its potential for intrauterine growth and development, and is typically caused by poor placental function [75,76]. Fetuses with FGR are at an increased risk of poor perinatal and neonatal outcomes, and they have higher rates of morbidity and mortality. In the absence of a diagnostic standard, a variety of metrics including fetal biometry, Doppler ultrasound, and small for gestational age (SGA), are used across studies to define FGR SGA describes fetal size at a given gestational age (e.g., below the 10th percentile) without considering the cause for small size or the growth trajectory in utero, and is commonly used as a surrogate for FGR [75,76]. However, most SGA infants do not show signs of placental dysfunction, nor are they at increased risk of adverse outcomes [77]. Therefore, discrepancies in reports of sex differences in FGR and SGA could be partly due to varying criteria, and using SGA as a surrogate for FGR could inflate the reported female risk of FGR.The threshold used to define SGA should be carefully considered.

For decades, female fetuses have been reported to be at an increased risk of FGR in several populations [55,57,73,78,79,80] (Table 2). Females also appear to be at higher risk of FGR in association with maternal hypertension [79], smoking [79,81], or asthma [82]. However, it is important to note that many of these studies use FGR interchangeably with SGA; only a few consider the presence of additional obstetric factors, or other metrics of serial ultrasonography. In a study using the head-to-abdominal circumference ratio, male fetal sex was identified as a risk factor for FGR only in women with a low pre-pregnancy weight and BMI [79]. Conversely, one study reported no sex differences in the incidence of preterm FGR [60]. It is possible that the risk of FGR in either sex may depend on additional factors, with males appearing more vulnerable to maternal anthropometric factors that limit fetal growth [79]. In addition, gestational age must also be considered, as early FGR (diagnosed < 32 weeks) is more often associated with abnormal Doppler studies and severe outcomes than late FGR (diagnosed > 32 weeks) [75].

While females can be over-diagnosed with SGA if using growth curves that are undifferentiated for sex, in studies using sex-specific growth curves, SGA females appear to be at a lower risk of experiencing adverse outcomes than SGA males [81]. SGA defined with sex-agnostic growth curves is less likely to reflect FGR or increased risk for other adverse outcomes [83,84], and may thus lead to unnecessary obstetric interventions, inadvertently increasing neonatal morbidity [83,85]. In addition, SGA defined with a fully customized fetal growth standard (adjusting for sex, parity, height, weight, and ethnicity) is associated with increased risk of poor outcomes [86]. 

### 2.4. Preeclampsia

Preeclampsia (PE) is commonly defined as maternal hypertension arising de novo after 20 weeks’ gestation accompanied by one or more adverse conditions, including proteinuria and/or maternal organ dysfunction [89,90]. The two most common clinical subtypes of PE are early-onset (EOPE) and late-onset (LOPE), depending on timing of diagnosis (prior to or at/after 34 weeks) [91,92]. While EOPE is more commonly associated with abnormal placentation, both forms are now thought to result from placental malperfusion, leading to syncytiotrophoblast damage [91,92]. Dividing PE into EOPE and LOPE at 34 weeks does not fully capture the spectrum of clinical, molecular, and pathophysiological features that vary across patients. This heterogeneity is important to consider when studying how sex affects PE, as illustrated by the conflicting results found in the literature.

Considering PE as a single entity irrespective of factors such as gestational age often reveals no differential incidence by sex [57,93,94], although sex differences have been reported in a few studies [95,96] (Table 3). More consistent sex differences are observed when stratifying PE by gestational age, with a female predominance in preterm PE (<37 weeks) [58,94]. A female excess is also observed in very preterm PE (<34 weeks) in several populations [69,94,97,98]. In contrast, either an equal sex ratio or slight male bias is reported for PE with term delivery (>37 weeks) [58,93]. The diversity of findings across studies highlights the importance of considering the heterogeneity of PE and gestational age when considering sex differences. Based on our current understanding, categorizing PE with variables such as gestational age, severity, or co-morbidities provides a more complete picture of sex differences in this disorder.

### 2.5. Stillbirth 

Stillbirth is most commonly defined as fetal death at or beyond 20 weeks of gestation or weight >500 g [99]. Some of the leading causes of stillbirth are asphyxia during labor, maternal factors, and placental dysfunction, which accounts for more than 50% of cases [100,101]. Unfortunately, most stillbirths occurring after 28 weeks of gestation are unexplained [101]. Male fetal sex has been recognized as one of the most prevalent risk factors for stillbirth [102]. A heightened male risk of perinatal morbidity and mortality is well reported in the literature [71,103,104,105,106], and a higher frequency of stillbirth among males has also been described [106,107,108]. However, nuances exist regarding male risk of stillbirth; for instance, one study noted that while male fetuses were at an increased risk of stillbirth, the association diminished with increasing birth weight quintile [103]. A few studies report no sex differences in the rates of stillbirth [71,104], while one study found female excess in stillbirths without any observed demographic or obstetric differences by sex at diagnosis [109]. In addition, a study of infant mortality in India and Pakistan, where the probable causes for stillbirth were similar in both male and female groups, revealed a significantly higher rate of male stillbirths and an increased risk for early perinatal mortality among male infants [107].

Findings are more variable for stillbirth coincident with other complications. There is an excess of males in stillbirths co-occurring with placental abruption [109,110], whereas an excess of females is observed for stillbirths associated with placental insufficiency or hypertension [109]. Sex differences in stillbirth risk are likely dependent on the underlying cause, and further research is needed to elucidate the role of fetoplacental sex as a risk factor for stillbirth.

## 3. Mechanisms for Sex Differences across Gestation

The cascade leading to phenotypic sex differences in both healthy and complicated pregnancies begins with the basic actions of sex chromosomes and steroid hormones (Table 4), which yield molecular consequences such as autosomal gene expression sex differences, and culminate in observable sex-specific phenotypes. Except in rare cases, the placenta harbours the same sex chromosome complement as the fetus and is subject to the effects of X and Y chromosome dosage disparities. Additionally, the fetoplacental unit produces hormones throughout gestation including estrogen, progesterone, and testosterone. Notable molecular consequences of prenatal sex differences include sex-specific patterns of gene expression, sex differences in key pregnancy hormones such as human chorionic gonadotropin, and sex differences in the fetoplacental response to maternal inflammation and infection. 

### 3.1. Sex Chromosome Effects 

#### 3.1.1. Peri-Implantation X Chromosome Dynamics 

Female-biased expression of X chromosome genes is one mechanism by which sex chromosomes may underlie phenotypic differences. In female (XX) mammals, one of the two X chromosomes is epigenetically silenced early in development by X-chromosome inactivation (XCI). XCI in humans occurs between implantation and tissue differentiation, and is completed approximately between 12 days and 1 month post-fertilization [111,112]. Prior to XCI, female X-linked genes are biallelically expressed as early as embryonic day three [112], and by embryonic day four, more than 25% of X-linked transcripts are expressed 2-fold higher in females [113]. It has been suggested that preimplantation growth differences are attributable to X chromosome effects, as male preimplantation embryos of several species exhibit faster metabolism and growth rates [114,115,116,117]. However, it is not yet clear whether sex-specific growth rates are also observed in vivo, and these observations may be artefacts of in vitro culture conditions [118,119]

#### 3.1.2. Escape from X-Chromosome Inactivation 

Following the establishment of XCI, cells of the female conceptus have one active and one inactive X chromosome. Though XCI dramatically reduces inactive X chromosome gene expression, up to 12% of genes escape XCI, and another 15% are reported to variably escape between tissues, individuals, or studies [120,121]. Genes that escape XCI are generally more highly expressed in females, though not always [120,122]. 

XCI escape genes in the placenta and fetus may contribute to phenotypic sex differences. DNA methylation is an epigenetic mark that assists with silencing gene expression on the inactive X [123,124]. Overall, DNA methylation levels are lower in the placental genome as compared to other tissues [125], and are specifically depleted on the placental inactive X chromosome [126]. Low placental inactive X DNA methylation may suggest that the placenta has a higher load of XCI escape genes than other tissues [126], which could widen the transcriptional gap between male and female placentae and contribute to phenotypic sex differences across gestation.

#### 3.1.3. Mosaic X-Chromosome Inactivation

In human embryonic and extraembryonic tissue, XCI is random and not imprinted via parent-of-origin; this in contrast to rodent extraembryonic lineages with paternally imprinted XCI. The human female placenta is thus a mosaic tissue often harbouring cell populations with a paternally active X chromosome and cell populations with a maternally active X chromosome [127,128]. Skewed XCI is the phenomenon by which >90% of cells within a tissue or individual inactivate the same parentally inherited X chromosome; skewed XCI in females can occur by chance, particularly if tissues are derived from a small pool of precursor cells or can occur if inactivation of one parental allele leads to a selective survival or proliferation advantage [129]. In placenta, such selection appears weak; instead due to clonal villous tree development there is a patchiness to XCI [130]. 

Aside from XY homologs in the pseudoautosomal regions, males have only a single copy of X chromosome genes and thus each X-linked variant in males has the potential to exert a greater phenotypic impact than in females [131]. Expression of mildly deleterious variants would have stronger effects in males [131] because they are constitutively expressed across the placenta, while the female placenta in theory could better moderate the effects of deleterious variants by the presence of some cell populations across the placenta inactivating the deleterious allele and limiting its impact.

#### 3.1.4. X Chromosome Dosage 

A more general effect of X chromosome biology on prenatal development is X chromosome dosage disparity by sex. Male (XY) and female (XX) cells differ in their typical X and Y chromosome complements. Several effects of X and Y chromosome dosage on prenatal development have been reported, though the precise mechanisms by which they act have not yet been elucidated. For example, presence of a single X chromosome has been associated with larger placentae in male compared to female pregnancies [3]. This effect replicates in mouse models where X chromosome dosage can be manipulated independently of phenotypic sex [132]; larger murine placentae were associated with offspring bearing a single X chromosome, independent of gonadal sex (male or female) and parental origin of the single X chromosome [133]. The precise mechanism by which X chromosome dosage affects placental size is not known but could involve any of the specific mechanisms described above.

#### 3.1.5. The Forgotten Y 

In addition to X chromosomal effects, the Y chromosome in male conceptuses may also drive sex differences. In the preimplantation period, 13 Y-linked genes are expressed at detectable levels [113], including four that lack X-linked homologs with similar function and are thus candidates for underlying phenotypic sex differences. Later in gestation, the mammalian sex-determining gene *SRY* is transcribed, and is critical for phenotypic masculinization [15]. Lack of *SRY* in males due to mutational events can in some cases result in gonadal dysgenesis or a disconnect between typical genotype and gonadal sex, as can *SRY* expression in females [134]. 

In other tissues, Y-linked genes have been found to contribute to autoimmune disease [135,136], likely owing to Y chromosome-encoded minor histocompatibility antigens (mHAgs) [137]. Y-linked mHAgs may also play a role in maternal immune tolerance of the male conceptus; at least six mHAgs are expressed in the human placenta, derived from the DDX3Y, KDM5D, and RPS4Y1 proteins [138]. Dysfunctional maternal immune tolerance of the fetus may therefore be sex specific, as women affected by RM secondary to one or more successful live births appear to be overrepresented for having a live born male preceding their recurrent losses [139,140]. This pattern has been independently confirmed [141], though a third study found no significant difference in the sex of the live birth preceding RM [142]. These women are also more likely to possess class II major histocompatibility antigens against Y-linked mHAgs, presumably arising from a maternal immune response to the preceding live born male [139]. A lower male/female birth ratio in subsequent live births has also been observed [139,143,144]. Together, these results suggest a Y-chromosomal contribution to sex biased pregnancy outcomes.

### 3.2. Steroid Hormone Effects 

#### 3.2.1. Estrogens and Progesterone 

Both male and female fetuses are exposed to high levels of estrogens throughout pregnancy, primarily in the form of estriol, with smaller contributions from estrone and estradiol [145]. Prenatally, estrone and estradiol are synthesized in the placenta from the fetal adrenal cortex-derived precursors dehydroepiandrosterone (DHEA) and dehydroepiandstrosterone sulfate (DHEA-S), while estriol is placentally synthesized from 16-*α*-hydroxyl DHEA-S arising in the fetal liver [146]. Prenatal levels of estriol and estradiol do not appear to differ by fetal sex [12,147], it is likely that estrone levels also do not differ by fetal sex, though studies are limited. DHEA levels also do not appear to differ by fetal sex [13,148], while the association of fetal sex and DHEA-S concentration has not been widely investigated. Estrogen is not be expected to be a major driver of prenatal sex differences, corroborated by evidence for normal fetal and placental growth in estrogen-deficient pregnancies [149]. However, a link exists between estrogen biology and prenatal complications. Estradiol promotes angiogenesis, vasodilation, and trophoblast proliferation/differentiation, processes which are compromised in PE [150]. A decrease in maternal blood estradiol, produced by the placenta, is also observed in pregnant women that subsequently develop PE [150,151]. Several genetic variants that decrease aromatase activity are associated with higher incidence of PE in a Japanese population, supporting an indirect mechanistic link between decreased estradiol production and PE [152]. 

Similar to estrogen, circulating fetal and maternal progesterone primarily derives from the placental syncytiotrophoblast [12]. Generally, progesterone is required for the maintenance of pregnancy and suppresses uterine contractility by direct inhibition of contraction-associated proteins in the myometrial tissue [153]. Amniotic fluid progesterone does not appear to differ by fetal sex in early or mid-gestation [12,13,154]. Though placental progesterone does not differ by sex, fetal response to maternal progesterone may: when progesterone is given to ovine mothers during early gestation, only male fetal progesterone concentration increases, apparently mediated by lower rates of progesterone metabolism in the male liver [155].

#### 3.2.2. Testosterone 

In uncomplicated gestations, prenatal androgens function to masculinize the male external genitalia approximately between the 8th and 16th weeks of gestation [12,13,156,157]. Masculinization is driven by fetal testosterone, mainly synthesized in the fetal adrenal cortex, testis, and the fetal ovary [12,158]. Androgen signalling occurs via the X-linked androgen receptor (AR) protein, loss of which leads to reduced male intrauterine growth in both mice and humans; variation in *AR* expression may also contribute to sex differences in fetal growth [159]. Fetal testosterone facilitates masculinization through its conversion to the more bioactive 5a-dihydroxytestosterone (DHT) upon reaching target organs [160]. However, a second and equally essential route to DHT relies on placental progesterone as an intermediate [13]. Placental insufficiency and FGR are frequently associated with abnormal external genital development in affected male offspring, possibly attributable to insufficient placental progesterone production [13]. 

Males experience maximum amniotic fluid testosterone concentrations between the 12th and 16th weeks of gestation [12,13,14]. At its peak, testosterone concentration is 2–5-fold higher in male amniotic fluid than in females [161,162,163,164], though there is overlap between the ranges observed in both sexes [13]. During this period of maximal sex difference in testosterone concentration, testosterone may establish the basis for sex-biased phenotypes. Beyond approximately 24 weeks of gestation until term, there are no significant sex differences in serum or amniotic fluid testosterone levels [12,13].

### 3.3. Molecular Consequences of Prenatal Sex Differences 

Though the effects of sex chromosomes and gonadal hormones are the basis of mammalian phenotypic sex differences, over the course of gestation there are notable downstream molecular consequences that are very sex divergent and likely have widespread impacts on development. Among the more immediate molecular consequences of either sex chromosome or sex hormone effects are widespread autosomal gene expression sex differences: up to 60% of sex-differentially expressed genes in the human placenta are autosomal [165,166]. Even during the preimplantation period, alongside X-linked expression differences, multiple autosomal genes (n = 58) are differentially expressed by sex [113]. Before the onset of fetal steroid hormone production, autosomal sex differences imply a relationship between sex chromosome dosage and autosomal gene expression. Though precise mechanisms of sex chromosome–autosome crosstalk in general are not yet clear, X chromosome effects have been somewhat explored and may be related to factors including X chromosome-encoded transcription factors, correlated networks of gene expression, or participation of autosomal genes in the process of XCI [167,168]. The epigenetically inactive X chromosome in each female nucleus also may impact autosomal gene regulation by acting as either a sink or source of epigenetic silencing factors [169]. 

While the levels of placentally synthesized steroid hormones do not tend to show sex biases, female-carrying pregnancies are associated with average higher maternal serum human chorionic gonadotropin (hCG) after the 3rd week of gestation, though precise male/female ratios vary across populations [170,171]. hCG is produced by the placenta, and the genes encoding the four hCG β subunits are among the most sex-differentially expressed placental autosomal genes [166]. hCG supports growth and invasion of the placenta, and regulates placental vascular endothelial growth factor (VEGF) and its receptors [172]. Though higher levels of hCG are observed in female-bearing pregnancies, females do not have larger placentas than males. This contradicts what one may expect if hCG promotes placental growth, and the reason for this apparent controversy is not yet understood. Additionally, while both the male fetus and placenta are larger than their female counterparts, there is a higher fetal/placental weight ratio in males, indicating that the male placenta is more efficient at promoting fetal growth [3].

Male and female placentae also exhibit marked differences in response to maternal glucocorticoid signalling, either endogenously derived or synthetically administered as antenatal betamethasone for expected preterm delivery [173,174]. In response to maternal glucocorticoid signalling, female fetal growth trajectories adaptively decrease due to alterations in placental glucocorticoid metabolism mediated by the 11β-hydroxysteroid dehydrogenase type 2 (11β-HSD2) enzyme, while male growth trajectories remain unchanged [173]. Higher levels of anti-inflammatory testosterone may protect males from the inflammatory effects of maternal glucocorticoid signaling elicit reduced growth [173], but also the lack of male adrenal adaptation to increased maternal glucocorticoid stimulation may leave males at a disadvantage in the face of preterm delivery [174].

Prenatal sex differences may also arise from sex differences in immunological function and response to inflammation. A higher rate of inflammation and infection is observed in male fetuses during intrauterine life, which may contribute to higher male perinatal mortality [4,175]. Chronic inflammation is more common in the decidua and basal plates of women carrying male offspring, suggesting greater maternal immune response to a male fetus [4]. Conversely, mothers carrying females exhibit greater stimulated cytokine production: across all trimesters, maternal serum levels of IL-6, IL-8, and TNF-a proinflammatory cytokines were significantly higher in association with a female fetus after PBMC lipopolysaccharide stimulation [176]. The cause of these differences is unknown, though, as discussed earlier, it is possible that some portion of the maternal immune response to the male fetus is mediated by Y chromosome antigens. 

## 4. Sex Differences in Diagnostic and Screening Approaches

Given the well-established sex differences in prenatal development, it is important that diagnostic and screening methods for pregnancy complications consider fetal sex and potentially optimize approaches separately for each sex. Sex-specific growth charts are a routinely-used tool, but other diagnostic approaches may similarly benefit from explicit consideration of sex.

There has been growing interest in the development of maternal serum screening tools for early diagnosis of pregnancy complication. However, the concentrations of many trophoblast-derived molecules assessed by such approaches may vary both by pathology and fetoplacental sex. For example, higher levels of two angiogenic factors involved in PE, soluble fms-like tyrosine kinase protein 1 (sFlt1) and placental growth factor (PLGF), are observed in maternal serum in association with a female fetus [177]. In terms of serum proteins evaluated prenatally, hCG and AFP are among several that differ by sex: the presence of a female fetus is associated with higher average maternal serum chorionic gonadotropin (hCG) after the 3rd week of gestation [170,171], and lower average second-trimester maternal serum alpha-fetoprotein (AFP) [178,179,180]. Female-carrying pregnancies are also associated with higher levels of maternal serum cell-free fetal DNA (cffDNA) [181], which is used for non-invasive prenatal testing (NIPT) of chromosomal abnormalities and fetal sex determination [181]. This cffDNA originates from trophoblast cells and represents 3–6% of the total cell-free circulating DNA in maternal circulation during gestation. Of note, cffDNA levels may correlate with the levels of serum proteins including hCG [182]. Maternal cervical fluid is also a source of trophoblast-derived nucleic acids; cervical fluid has valuable diagnostic potential and can be used to accurately assess fetal sex [183]. It is possible that the interactions of sex and trophoblast-derived markers in cervical fluid may differ from those measured in maternal serum. These examples illustrate the interdependence of biomarker species with fetoplacental sex.

Sex should especially be considered when phenotypes, etiologies, or prognostic markers are a priori known to interact with sex. For example, elevated hCG and low AFP are both observed in association with female offspring, and separately, are indicative of elevated risk of aneuploidy [184]. Though there does not appear to be a bias for higher hCG positive screen rates in females [178,185], an excess in female positive screens is observed for AFP [186]. As AFP and hCG in combination with other factors may also be prognostic for PTB, FGR, and/or PE, sex should be of special consideration given its association with both the markers of interest and the disorders themselves [177,187]. Maternal serum sFlt1/PlGF ratios have been proposed for predicting PE and FGR [188,189,190,191]. However, higher levels of maternal serum sFlt1 in female-bearing pregnancies should elevate sFlt1/PIGF ratios [191], while conflicting reports suggest PlGF may also vary with fetal sex [177,192]. The effect of fetal sex on sFlt1/PlGF ratios should be carefully elucidated in both healthy gestations and in the context of PE, to understand the interaction of sex and pathology on this ratio prior to effective clinical implementation [177]. An increase in maternal plasma leptin was observed in EOPE pregnancies carrying a male fetus, suggesting an interaction between sex and PE [193]. More research is needed to evaluate how sex affects the diagnostic utility of such biomarkers.

During pregnancy, fetal sex can be assigned genetically (by NIPT, chorionic villus sampling, or amniotic fluid sampling) or anatomically via ultrasound. Depending on the driver of particular sex differences (sex chromosomes or gonadal hormones), as well as their timing and persistence, different sex assessment methods may prove differentially valuable. Additionally, while sex is typically considered a binary trait, research has illustrated high degrees of variability and overlap in many sex-related phenotypes. Specifically, neuropsychiatric research has begun to adopt a model of sex differences that range from defining male–female differences as sexually dimorphic (categorically distinct and not overlapping) to sex differences with continuous endpoints, to differences with the same endpoint achieved by distinct mechanisms in each sex [194]. The state of this field is reviewed in [195,196,197].

Prenatal diagnostics should also consider ethnicity or ancestry, as healthy phenotypes in one population could be labelled pathogenic if measured with standards developed in another. When considering genomic screening methods, one must consider the frequency at which “risk” variants exist in certain populations. For example, it has been reported that an *IL6* variant associated with acute chorioamnionitis risk is only present in East Asian populations [198], and that selection in the progesterone receptor gene may have led to specific polymorphisms that underlie differential rates of progesterone-associated pregnancy complications by population [199]. Other maternal factors associated with risk of adverse pregnancy outcomes include socioeconomic status, comorbid health conditions, and smoking status. Some of these factors are well known to interact with sex and are therefore of particular importance to consider; for example, both socioeconomic status and maternal asthma influence maternal glucocorticoid signaling, known to elicit different fetal responses based on sex [82,200].

## 5. Conclusions

Epidemiological studies have revealed sex differences associated with the incidence and outcomes of several obstetric complications, with the widespread claim of a “male disadvantage” being more nuanced than initially thought, and dependent on additional variables such as gestational age. These findings have warranted further study into the biological mechanisms underlying prenatal sex differences, which we must continue to elucidate if fetal sex is to be incorporated into clinical consideration in the context of diagnostic tests and interventions. As clinical practice is steadily evolving towards precision medicine initiatives, prenatal care will follow suit, and it is clear that the evidence suggests it will be of value for researchers and clinicians to consider how proper integration of sex considerations can improve current or future diagnostic methods for pregnancy complications. Lastly, incorporating fetoplacental sex in diagnosis and screening should not obscure the equal importance of other variables such as genetic ancestry, which are also extremely relevant to perinatal health and may in fact interact with sex in many contexts.

## Figures and Tables

**Table 1:** Summary of findings from preterm birth (PTB) studies. An asterisk (*) indicates n_PTB_ < 1000.

**M/F Ratio > 1 (Male Predominance)**	**Population(s)**	**Reference**
High in spontaneous PTB (not in induced PTB or with any antenatal pathology) Low at/after term	Aberdeen, UK	[50]
High in PTB among white singleton births Balanced in Black singleton births	New England, US	[51]
High in PTB compared to term births up to 37 w	Italy	[52]
High in PTB; particularly in early and spontaneous PTB Balanced in two cohorts of PTB Black singleton births, induced PTB, and spontaneous PTB after IVF	Europe	[54]
High in PTB; males account for 55% of all newborns at 23–32 w High in neonatal mortality, particularly at early GA	Sweden	[71]
High in spontaneous PTB Balanced in induced PTB Low in early PTB with hypertension	France	[60]
High in spontaneous PTB High in perinatal mortality throughout pregnancy Low in PTB with preeclampsia	Norway	[58]
High in spontaneous PTB Low in induced PTB	Oxford, UK	[59]
High in spontaneous PTB between 34 and 36 w but not <34 w, and after adjustment for confounding factors	Southern China	[72]
High in PTB and PPROM, even after adjusting for fetal weight	Spain	[57]
High in PTB even after controlling for birth weight	Libya	[69]
High in PTB even after adjustment for cofounders including hospital grade, maternal age, bad obstetric history, and other medical disorders	Mainland China	[73]
High in spontaneous PTB with intact membranes and with PPROM, with a more pronounced effect in PTB at <32 w	Netherlands	[56]
High in preterm labor and PTB Balanced in preterm labor and PTB in non-Caucasian women	Netherlands	[74] *
High in spontaneous and iatrogenic PTB, although iatrogenic PTB shows a bias for either sex depending on the statistical method used	South Australia	[62]
High in PTB in an African, Asian and Mediterranean population, although the population-attributable risk of male fetal sex on spontaneous PTB was lowest in African women and highest in Mediterranean women	African, Asian and Mediterranean	[70]
**M/F Ratio < 1 (Female Predominance)**	**Population (s)**	**Reference**
Low in PTB	Indigenous Australian	[68]
Low in spontaneous and iatrogenic PTB in a cohort of high-risk women for PTB	White, Black, South Asian, and Other	[67]
Low in iatrogenic PTB	Belgium	[61] *

**Table 2:** Summary of findings from fetal growth restriction (FGR) and small for gestational age (SGA) studies, PTB indicates pre-term birth. An asterisk (*) indicates n_FGR_ < 1000 or n_SGA_ < 1000.

Criteria Used to Define FGR/SGA	Main Findings	Population(s)	Reference
**Female Predominance**			
BW < 10th percentile for GA, included some studies with <2500 g birth weight plus GA > 37 w.	Male fetuses have a higher BW and lower risk of SGA across all populations studied. Female fetal sex is more significantly associated with SGA in developed countries.	North America, Western Europe, Africa, Latin America, Southeast Asia, India	[78]
BW < 10th percentile for GA.	Female fetuses at higher risk of SGA.	Lebanon	[80]
BW and GA < 10th percentile.	Higher female risk for SGA with maternal smoking.	Germany	[81]
Unspecified.	Greater incidence of SGA among female fetuses, independent of other SGA risk factors such as preeclampsia.	Israel	[55]
Echographic diagnosis (criteria unspecified).	FGR more frequent among female fetuses.	Spain	[57]
BW < 10th percentile.	SGA more frequent among female fetuses.	Mainland China	[73]
Suspicion of FGR based on poor fetal growth for BW percentile, and presence of obstetric risk factors.	Females more often suspected of FGR according to risk factors for SGA infants with a birthweight <10th and <3rd percentile.	France	[85]
Serial ultrasonography (SU); increase in the head-to-abdominal circumference ratio up to >2 SDs above the mean, or failure of either abdominal or head circumference to grow on 2 consecutive examinations 2 w apart.	FGR more frequent among females according to SU and SGA curves. Female risk higher with maternal hypertension and smoking. Male risk higher with low maternal pre-pregnancy weight and BMI.	Italy	[79] *
**No Effect or Male Predominance**			
Ethnicity- and sex-specific BW < 10th percentile for GA.	FGR slightly more frequent in males FGR males at higher risk of all adverse outcomes studied, including neonatal death, necrotizing enterocolitis, and respiratory distress syndrome.	Vermont (white and African American)	[87]
Unspecified.	No male excess among PTB associated with FGR.	France	[60]
BW < 10th percentile for GA.	No differences in SGA outcomes by sex. Fetal sex not an independent risk factor for adverse outcomes in SGA.	Pennsylvania, US	[88] *

**Table 3:** Summary of findings from preeclampsia (PE) studies. An asterisk indicates n_PE_ < 1000.

Main Findings	Population(s)	Reference
**Male Predominance**		
Slightly more male pregnancies with PE (not stratified for gestational age).	Denmark	[96]
Male preponderance in PE, No significant sex differences in any of the studied obstetric complications usually secondary to PE, including placental abruption, placenta previa, and stillbirth.	Missouri, US	[95]
**Female Predominance in Preterm PE**		
Preterm PE (<37 weeks) more frequent among females. Sex ratio reversed >37 weeks, male fetal sex associated with PE. 40–42 weeks, equal proportion of males and females with PE.	Norway	[58]
Compared to all infants born <32 weeks, those with PE <32 weeks more often female. At term, the M/F ratio is increased in PE.	Sweden	[93]
Female singleton pregnancies had increased incidence of PE. Female–female monochorionic diamniotic (MD) and dichorionic diamniotic (DD) pregnancies had a higher incidence of PE than their male counterparts in both MD and DD pregnancies, respectively.	Japan	[98]
Overall incidence of PE not associated with fetal sex. Preterm PE more common in pregnancies carrying a female fetus, even after adjustment for confounders.	Northern China	[97]
No sex difference in incidence of PE (not stratified for GA). Female fetal sex associated with preterm PE. Post-term PE more frequent among male fetuses. Male fetuses of primigravid women had a greater likelihood of developing PE than female-bearing primigravid women.	Libya	[69] *
No sex differences in all PE, term PE (>37 w), and PE 34–37 w. Female predominance in very preterm (<34 w) PE.	Europe, US, New Zealand, Australia	[94]
**No Sex Differences**		
No sex differences in incidence of PE (not stratified for GA).	Spain	[57]

**Table 4:** Mechanisms underlying sex differences across gestation, XCI indicates X-chromosome inactivation.

Mechanism	Description
Escape from XCI	Genes that escape XCI may be more highly expressed in females. Proportion of XCI escape in placenta may be greater than other somatic tissues.
Mosaicism for XCI	Patterns of XCI across placenta (mosaicism for parental inactive X) may enable females to better tolerate deleterious alleles.
X chromosome dosage	Before implantation, females have two active X chromosomes. During this period, X-linked genes are biallelically and more highly expressed in female cells. Coincident autosomal gene expression sex differences observed. Single X chromosome associated with larger placentae at term (in humans; in mice this holds true and is independent of gonadal steroids).
Y chromosome	Preimplantation expression of Y-linked genes in XY embryos. Y chromosome minor histocompatibility antigens in placenta may interact with maternal immune system to mediate perinatal complications including secondary RM.
Estrogen and progesterone	Amniotic fluid levels not reported to differ by sex, likely do not have strong influence on sex-biased phenotypes.
Testosterone	Initially synthesized mid-late first trimester, peak concentration in male amniotic fluid 12–16 weeks’ gestation and is 2–5-fold higher than observed in females. Has the potential to contribute to sex-biased phenotypes

## Data Availability

Not applicable.
